# Spexin as an indicator of beneficial effects of exercise in human obesity and diabetes

**DOI:** 10.1038/s41598-020-67624-z

**Published:** 2020-06-30

**Authors:** Abdelkrim Khadir, Sina Kavalakatt, Dhanya Madhu, Sriraman Devarajan, Jehad Abubaker, Fahd Al-Mulla, Ali Tiss

**Affiliations:** 1 Biochemistry and Molecular Biology Department, Research Division, Dasman Diabetes Institute, P.O. Box1180, 15462 Dasman, Kuwait; 2 Research Division, Dasman Diabetes Institute, Dasman, Kuwait

**Keywords:** Biomarkers, Diseases

## Abstract

Spexin is a novel neuropeptide playing an emerging role in metabolic diseases such as obesity and diabetes via involvement in energy homeostasis and food intake. The present study investigated the effects of obesity and type 2 diabetes (T2D) on circulating levels of spexin and its modulation by physical exercise. Normal-weight (n = 50) and obese adults with and without T2D (n = 69 and n = 66, respectively) were enrolled in the study. A subgroup of obese participants (n = 47) underwent a supervised 3-month exercise programme. Plasma spexin levels were measured by ELISA and correlated with various markers. Plasma spexin levels decreased in obese participants with or without T2D compared with those of normal-weight participants (0.43 ± 0.11, 0.44 ± 0.12 and 0.61 ± 0.23 ng/ml, respectively; *P* < 0.001). Spexin levels negatively correlated with adiposity markers and blood pressure in the whole study population (*P* < 0.05). Multiple regression analysis revealed blood pressure was the greatest predictive determinant of plasma spexin levels, which significantly increased in response to physical exercise in obese participants without and with T2D (*P* < 0.05). Spexin levels significantly increased only in responders to exercise (those with increased oxygen consumption, VO_2_ max) with a concomitant improvement in metabolic profile. In conclusion, plasma spexin levels may be an indicator of response to physical exercise.

## Introduction

Energy homeostasis is regulated by complex communication pathways between the brain and peripheral organs, and disruption of these pathways significantly contributes to chronic metabolic diseases such as obesity and diabetes^1^. The hypothalamus produces neuropeptides that are involved in energy homeostasis. For example, genetic forms of obesity involve neuropeptides such as melanocortin-4-receptor deficiency^2^. Although these neuropeptides were first discovered in the brain, many are also expressed in various peripheral metabolic organs such as skeletal muscle, the pancreas and adipose tissue^3^. Molecules that mimic the action of neuropeptides are currently being developed for the treatment of obesity^4^.

Spexin, also known as neuropeptide Q, is a recently identified neuropeptide that is thought to be involved in energy homeostasis. It was primarily discovered using bioinformatics search strategies^5^. Studies using rodents and goldfish have shown the presence of spexin mRNA and protein in several tissues, including hypothalamus, hippocampus, cerebral cortex, stomach, kidney, adrenal gland and ovaries^6^. Injection of spexin into goldfish and a diet-induced obesity rodent model decreased food intake, which was linked to downregulation of orexigenic factors and upregulation of anorexigenic factors^7,8^. On the other hand, blood spexin levels were decreased in both adults and children with obesity and diabetes^6^, although another study showed no difference in serum spexin levels between three groups of adolescents (normal-weight, obese and obese with diabetes)^9^. Moreover, circulating spexin levels displayed a borderline association with components of metabolic syndrome in adults^10^. Spexin also contributes to central nervous system-mediated modulation of nociceptive responses^11^ and, like other neuropeptides, may also play a role in pain control. These neuropeptides were also associated with inherent differences and alterations in pain control between ethnic groups^12^.

Spexin gene expression was downregulated in the adipose tissue of obese patients compared with that of normal-weight controls as well as between heavy and lean co-twins^8,13^. A 1-year longitudinal study conducted in patients following Roux-en-Y gastric bypass (RYGB) in young adults with severe obesity reported increased levels of circulating spexin post-surgery^14^. Another nonpharmacological strategy widely used to manage obesity and its related complications is regular physical exercise as a component of a healthy lifestyle. Exercise has pleiotropic effects on physiology by reducing food intake and body weight^15^ as well as altering neuropeptide levels, particularly orexigenic and anorexigenic peptides^16^, thus improving obesity-related conditions. A recent study reported favourable effects of a 6-month self-monitored lifestyle modification programme that included physical exercise on circulating spexin levels in females with prediabetes^17^.

Taken together, these findings suggest that spexin plays a critical role in metabolic homeostasis and is potentially modulated by physical exercise. Since spexin is presumed to play a role in regulating body weight, we compared levels of circulating spexin between normal-weight, obese and obese patients with diabetes in an Arab population and evaluated the association of these levels with demographic and clinical parameters. We also investigated whether physical exercise could influence spexin levels in obese patients. Our findings indicated that levels of spexin were reduced with obesity and diabetes but increased in response to physical exercise.

## Results

### Plasma spexin levels are dysregulated in obesity and diabetes

Anthropometric, clinical and biochemical parameters are summarised in Table 1. The obese with diabetes group was older compared with the obese without diabetes and normal-weight groups (*P* < 0.001). Both the obese groups (with and without diabetes) displayed significantly higher blood pressure and hsCRP levels but lower VO_2_ max levels compared with the normal-weight group (*P* < 0.01). Moreover, the obese with diabetes group showed a more dysregulated lipid profile, as indicated by lower high-density lipoprotein (HDL) (*P* < 0.001) and higher TG (*P* < 0.001) levels, compared with the normal-weight and obese without diabetes groups. Both obese groups showed higher levels of glycemic markers (fasting blood glucose (FBG), HbA1c and insulin) (*P* < 0.05) compared with the normal-weight group (Table 1).

**Table 1:** Physical, clinical and biochemical characteristics of the study population.

	Normal weight^a^ (N = 50)	Obese without diabetes^b^ (N = 66)	Obese with diabetes^c^ (N = 69)	P-trend	*P* value ^a^ versus ^b^	*P* value ^a^ versus ^c^	*P* value ^b^ versus ^c^
Gender (M/F)	20/30	27/38	36/33	0.304			
Age (years)	39.96 ± 11.31	43.22 ± 12.44	52.11 ± 9.43	< 0.001	0.169	< 0.001	< 0.001
BMI (kg/m^2^)	23.07 ± 2.14	34.55 ± 3.13	34.23 ± 2.56	< 0.001	< 0.001	< 0.001	1.000
Waist (cm)	81.77 ± 9.86	107.25 ± 11.11	110.19 ± 8.63	< 0.001	< 0.001	< 0.001	0.184
Hip (cm)	95.28 ± 14.64	116.53 ± 11.90	114.60 ± 12.10	< 0.001	< 0.001	< 0.001	0.982
PBF (%)	29.08 ± 5.39	38.55 ± 5.51	38.37 ± 8.56	< 0.001	< 0.001	< 0.001	1.000
WBC10	6.25 ± 1.46	6.54 ± 1.74	7.43 ± 1.82	< 0.001	0.832	< 0.001	0.001
SBP (mmHg)	111.64 ± 10.37	120.21 ± 11.54	122.59 ± 11.43	< 0.001	< 0.001	< 0.001	0.518
DBP (mmHg)	73.94 ± 6.59	77.63 ± 7.64	78.73 ± 6.28	0.001	0.041	0.001	0.913
Resting HR (beats/min)	81.68 ± 11.50	78.88 ± 10.99	82.41 ± 13.26	0.037	0.239	1.000	0.056
VO_2_, max (ml/kg/min)	21.23 ± 5.08	17.45 ± 4.45	15.31 ± 4.07	< 0.001	< 0.001	< 0.001	0.007
TC (mmol/l)	5.10 ± 0.96	5.16 ± 0.96	4.96 ± 1.15	0.365	1.000	1.000	0.481
HDL (mmol/l)	1.44 ± 0.48	1.22 ± 0.31	1.17 ± 0.39	< 0.001	0.001	< 0.001	1.000
LDL (mmol/l)	3.13 ± 0.85	3.32 ± 0.89	2.99 ± 1.26	0.085	0.751	1.000	0.082
TG (mmol/l)	0.97 ± 0.66	1.37 ± 0.81	1.82 ± 1.17	< 0.001	0.026	< 0.001	0.002
FBG (mmol/l)	5.05 ± 0.58	5.46 ± 0.94	9.18 ± 3.57	< 0.001	0.738	< 0.001	< 0.001
HbA1c (%)	5.51 ± 0.43	5.77 ± 0.88	8.41 ± 1.84	< 0.001	0.545	< 0.001	< 0.001
Insulin (ng/ml)	2.81 ± 1.26	3.89 ± 2.42	4.01 ± 2.05	0.007	0.023	0.010	1.000
C-peptide (ng/ml)	3.86 ± 4.53	5.35 ± 6.41	4.49 ± 5.93	0.418	0.589	1.000	1.000
HOMA-IR	0.63 ± 0.28	0.95 ± 0.63	1.50 ± 0.89	< 0.001	0.063	< 0.001	< 0.001
hsCRP (μg/ml)	3.45 ± 4.39	5.97 ± 4.50	5.78 ± 4.35	0.003	0.02	0.03	0.879

Data are presented as the mean ± SD.

*BMI* body mass index, *PBF* percent body fat, *SBP* systolic blood pressure, *DBP* diastolic blood pressure, *HR* heart rate, *VO*_*2*_* max* maximum oxygen consumption, *TC* total cholesterol, *HDL* high-density lipoprotein, *LDL* low-density lipoprotein, *TG* triglyceride, *FBG* fasting blood glucose, *HbA1c* hemoglobin A1c, *HOMA-IR* Homeostatic Model Assessment for Insulin Resistance, *hsCRP* high-sensitivity C-reactive protein.

^a^Normal weight group

^b^Obese without diabetes group

^c^Obese with diabetes group.

**Figure 1:**
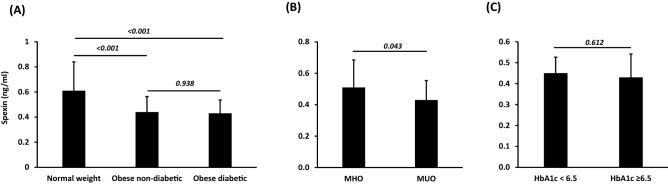
Spexin levels in the plasma. (**A**) Circulating levels of spexin were measured by ELISA using plasma samples from normal-weight (n = 50) and obese people without (n = 66) and with diabetes (n = 69) at baseline. 2-way ANOVA test was used to determine significance of difference in means between the groups. (**B**) Obese participants without diabetes were segregated into 2 groups, metabolically healthy obese (MHO) and metabolically unhealthy obese (MUO) based on the Adult Treatment Panel-III (NCEP-ATPIII guideline) criteria for metabolic syndrome components^18^ including the following criteria: (1) TG ≥ 150 mg/dl; (2) HDL-C < 40 mg/dl for men and < 50 mg/dl for women; (3) BP ≥ 130/85 mm Hg and (4) FBG ≥ 100 mg/dl. Nonparametric Wilcoxon test was used to determine significance of difference in means between the two groups. (**C**) Good and poor glycaemic control participant classification are based on HbA1c levels. Nonparametric Wilcoxon test was used to determine significance of difference in means between the two groups.

Plasma spexin levels were significantly lower in both the obese groups (*P* < 0.001) (Fig. 1A); however, there were no differences in levels between these two groups (*P* = 0.938). Not all obese individuals develop metabolic dysregulation and diabetes, and such individuals are classified as metabolically healthy individuals, despite increased body mass index (BMI). Likewise, not all non-obese individuals present a healthy metabolic profile. We divided our obese without diabetes group into metabolically healthy obese (MHO) and metabolically unhealthy obese (MUO) groups on the basis of previously reported criteria^18^ and found that levels of spexin were significantly lower in the MUO group (*P* = 0.043) (Fig. 1B). However, spexin levels were similar (*P* = 0.612) when we further divided the obese with diabetes group on the basis of HbA1c levels, controlled (HbA1c < 6.5%) and uncontrolled (HbA1c ≥ 6.5%). The clinical and demographic characteristics of these two subcohorts are shown in Tables S1 and S2.

Spearman correlation analysis of plasma spexin levels with various demographic and clinical parameters was performed for all participants as well as separately in the normal-weight, obese without diabetes and obese with diabetes groups (Table 2). Spexin levels were inversely correlated with adiposity markers in the whole population (BMI and waist and hip circumferences) as well as with waist circumference in the normal-weight and obese with diabetes groups. Moreover, analysis of all the study participants showed that spexin levels correlated negatively with blood pressure but positively with HDL. Within the normal-weight group, there was a significant positive correlation of spexin with VO_2_ max and WBC10, whereas it correlated negatively with diastolic blood pressure (DBP) and HbA1c. This correlation differed among obese participants depending on the presence or absence of diabetes. In the obese without diabetes group, spexin levels were negatively correlated with cholesterol and low-density lipoprotein (LDL) but positively correlated with HOMA-IR. On the other hand, spexin levels were positively correlated with HDL but negatively correlated with TG levels in the obese with diabetes group.

**Table 2:** Spearman correlation of circulating spexin with population characteristics.

	All	Normal weight	Obese without diabetes	Obese with diabetes
Age (years)	0.004	− 0.119	− 0.037	0.057
BMI (kg/m^2^)	− 0.151*	− 0.028	0.155	0.044
Waist (cm)	− 0.241**	− 0.398*	0.207	− 0.324*
Hip (cm)	− 0.257**	− 0.207	0.010	0.012
PBF (%)	− 0.133	− 0.050	0.085	0.237
WBC10	0.129*	0.365*	0.165	0.064
SBP (mmHg)	− 0.163*	− 0.363*	0.027	− 0.202
DBP (mmHg)	− 0.163*	− 0.385*	0.204	− 0.256
Resting HR (beats/min)	− 0.023	0.136	0.053	− 0.130
VO_2_, max (ml/kg/min)	0.122	0.382*	0.080	− 0.195
TC (mmol/l)	− 0.105	0.070	− 0.384**	− 0.076
HDL (mmol/l)	0.131*	0.099	− 0.062	0.388**
LDL (mmol/l)	− 0.112	0.140	− 0.406**	− 0.131
TG (mmol/l)	− 0.078	0.013	0.005	− 0.248*
FBG (mmol/l)	− 0.027	− 0.213	0.175	− 0.094
HbA1c (%)	− 0.097	− 0.420**	− 0.092	0.028
Insulin (ng/ml)	0.014	0.253	0.232	− 0.092
C-peptide (ng/ml)	0.066	0.110	0.071	0.028
HOMA-IR	− 0.019	0.120	0.266*	− 0.156
hsCRP (μg/ml)	− 0.127	0.345	− 0.074	− 0.205

*BMI* body mass index, *PBF* percent body fat, *SBP* systolic blood pressure, *DBP* diastolic blood pressure, *HR* heart rate, *VO*_*2*_*, max* maximum oxygen consumption, *TC* total cholesterol, *HDL* high-density lipoprotein, *LDL* low-density lipoprotein, *TG* triglyceride, *FBG* fasting blood glucose, *HbA1c* hemoglobin A1c, *HOMA-IR* Homeostatic Model Assessment for Insulin Resistance, *hsCRP* high-sensitivity C-reactive protein.

Significance of spearman correlation are as follows *P < 0.05, **P < 0.01.

Multivariate stepwise linear regression analysis was performed with spexin as a dependent variable in the whole study population as well as separately in the normal-weight, obese without diabetes and obese with diabetes groups using Spearman correlated variables (Table 3). In the entire population and the normal-weight group, only DBP was an independent determinant of spexin levels (*P* < 0.05). However, in the obese without diabetes group, negative independent associations were observed between spexin levels and LDL and HOMA-IR (*P* < 0.01). In the obese with diabetes group, only HDL was independently associated with circulating spexin levels (*P* < 0.01).

**Table 3:** Multivariate linear regression analysis with spexin as dependent variable.

Independent variables	All		Normal weight		Obese without diabetes		Obese with diabetes	
	β coefficient	*P* value	β coefficient	*P* value	β coefficient	*P* value	β coefficient	*P* value
DBP	− 0.160	0.040	− 0.614	0.003				
LDL					− 0.421	< 0.001	–	–
HOMA-IR					− 0.308	0.009	–	–
HDL					–	–	0.375	0.002

Adjusted for age, gender and BMI.

### Physical exercise increased spexin levels in the obese groups irrespective of diabetes

**Figure 2:**
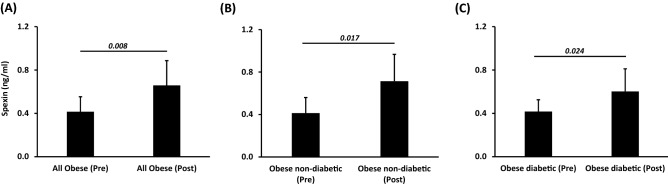
Physical exercise increased the release of spexin in obese participants. Circulating levels of Spexin were measured by ELISA using plasma samples from (**A**) all obese participants, or obese participants without (**B**) and (**C**) with diabetes before and after a 3-month physical exercise intervention (n = 47, n = 20 and n = 27, respectively). The *P* value was determined using a paired *t*-test for intragroup comparisons before and after exercise.

**Figure 3:**
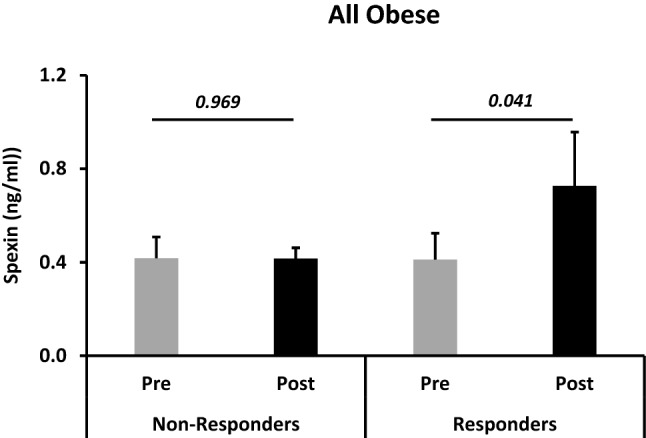
Spexin levels are increased in obese participants displaying improvement of VO_2_ max due to exercise. Circulating levels of Spexin were measured by ELISA using plasma samples from obese people and segregated according to increased (responders, n = 33) or not (non-responders, n = 14) VO_2_ max levels after 3-month physical exercise. The *P* value was determined using a paired *t*-test for intragroup comparisons before and after exercise.

We assessed the effects of physical exercise by performing a pairwise comparison before and after the 3-month exercise programme of spexin levels as well as clinical and metabolic parameters on a subset of our study population [obese without diabetes (n = 20) and obese with diabetes (n = 27)]. In both obese groups, exercise significantly increased blood spexin levels (*P* < 0.05) with concurrent amelioration of clinical and metabolic markers (Fig. 2). In the obese without diabetes group, a statistically significant increase in VO_2_ max and decreased percent body fat and insulin levels were observed (Table 4). However, a significant improvement in heart rate (HR) with concomitant decreased levels of HbA1c and total cholesterol (TC) was observed after exercise in the obese with diabetes group (Table 5). We further performed Spearman correlation analysis for HR, VO_2_ max and Spexin with the other characteristics of the individuals enrolled into the exercise protocol (n = 47) before and after exercise (Table S3). While Spexin levels were inversely correlated with TG before exercise, they further inversely correlated with Hip circumference, insulin, HOMA-IR and hsCRP levels. As expected, HR and VO2 max inversly correlated after exercise in those individuals. Furthermore, when the two obese groups (with and without diabetes) were clustered on the basis of amelioration of cardiorespiratory fitness VO_2_ max after exercise (Table 6), we observed that spexin levels were only significantly increased in responders (individuals with increased VO_2_ max value after exercise as compared to their VO_2_ max before exercise), with decreased HR and hsCRP levels (Fig. 3).

**Table 4:** Characteristics of obese without diabetes participants before and after exercise (n = 20).

	Obese without diabetes (pre)	Obese without diabetes (post)	Variation	*P* values
BMI (kg/m^2^)	34.3 ± 3.0	33.9 ± 3.8	− 0.44 ± 0.64	0.196
Waist (cm)	104.0 ± 7.6	101.0 ± 6.2	− 2.93 ± 2.24	0.102
Hip (cm)	114.8 ± 8.2	111.7 ± 9.3	− 3.05 ± 2.17	0.135
PBF (%)	39.0 ± 4.4	37.8 ± 5.5	− 1.21 ± 1.77	0.037
WBC10	5.9 ± 1.9	5.7 ± 1.7	− 0.17 ± 1.18	0.627
SBP (mmHg)	115.0 ± 8.2	114.5 ± 6.9	− 0.50 ± 3.44	0.627
DBP (mmHg)	75.0 ± 5.3	75.0 ± 5.8	0.03 ± 1.82	1.000
Resting HR (beats/min)	80.3 ± 8.7	78.6 ± 13.6	− 1.70 ± 9.78	0.596
VO_2_, max (ml/kg/min)	17.1 ± 3.7	19.8 ± 4.9	2.65 ± 3.15	0.026
TC (mmol/l)	5.2 ± 1.1	5.1 ± 1.2	− 0.11 ± 0.68	0.499
HDL (mmol/l)	1.3 ± 0.3	1.3 ± 0.5	− 0.05 ± 0.26	0.434
LDL (mmol/l)	3.5 ± 1.0	3.2 ± 1.0	− 0.24 ± 0.64	0.127
TG (mmol/l)	1.1 ± 0.4	1.3 ± 0.9	0.20 ± 0.87	0.159
FBG (mmol/l)	5.3 ± 0.8	5.5 ± 1.1	0.16 ± 0.64	0.308
HbA1c (%)	5.9 ± 1.7	5.7 ± 0.5	− 0.20 ± 1.28	0.201
Insulin (ng/ml)	4.1 ± 2.7	3.2 ± 1.6	− 1.06 ± 2.63	0.039
C-peptide (ng/ml)	8.8 ± 12.1	6.3 ± 7.2	− 2.53 ± 3.75	0.138
HOMA-IR	1.1 ± 0.6	0.8 ± 0.2	− 0.34 ± 0.75	0.070
hsCRP (μg/ml)	5.2 ± 0.9	5.7 ± 1.7	0.52 ± 2.93	0.090

Data are presented as the mean ± SD.

Paired t-test was used to compare differences in participants before and after physical exercise.

*BMI* body mass index, *PBF* percent body fat, *SBP* systolic blood pressure, *DBP* diastolic blood pressure, *HR* heart rate, *VO*_*2*_*, max* maximum oxygen consumption, *TC* total cholesterol, *HDL* high-density lipoprotein, *LDL* low-density lipoprotein, *TG* triglyceride, *FBG* fasting blood glucose, *HbA1c* hemoglobin A1c, *HOMA-IR* Homeostatic Model Assessment for Insulin Resistance, *hsCRP* high-sensitivity C-reactive protein.

**Table 5:** Characteristics of obese with diabetes participants before and after exercise (n = 27).

	Obese with diabetes (pre)	Obese with diabetes (post)	Variation	*P* values
BMI (kg/m^2^)	33.8 ± 2.4	33.1 ± 3.6	− 0.71 ± 0.59	0.356
Waist (cm)	111.8 ± 5.1	109.5 ± 5.8	− 2.32 ± 2.13	0.115
Hip (cm)	113.5 ± 6.1	113.5 ± 5.6	− 0.01 ± 0.19	0.988
PBF (%)	38.3 ± 5.1	36.1 ± 5.3	− 2.18 ± 1.56	0.055
WBC10	7.6 ± 1.3	7.4 ± 1.8	− 0.23 ± 0.09	0.622
SBP (mmHg)	122.2 ± 13.0	122.6 ± 8.6	0.41 ± 1.38	0.908
DBP (mmHg)	76.7 ± 5.0	75.8 ± 5.7	− 0.89 ± 0.47	0.769
Resting HR (beats/min)	86.0 ± 7.0	78.0 ± 11.7	− 8.01 ± 6.83	0.042
VO_2_, max (ml/kg/min)	16.7 ± 4.2	20.1 ± 4.9	3.41 ± 3.89	0.173
TC (mmol/l)	4.8 ± 1.1	4.4 ± 0.9	− 0.38 ± 0.29	0.041
HDL (mmol/l)	1.1 ± 0.3	1.0 ± 0.3	− 0.07 ± 0.17	0.165
LDL (mmol/l)	2.9 ± 1.1	2.8 ± 0.9	− 0.11 ± 0.24	0.702
TG (mmol/l)	1.7 ± 0.5	1.6 ± 0.8	− 0.09 ± 0.28	0.799
FBG (mmol/l)	8.6 ± 3.4	8.2 ± 3.2	− 0.41 ± 0.88	0.515
HbA1c (%)	8.2 ± 2.0	7.3 ± 1.3	− 0.85 ± 0.63	< 0.001
Insulin (ng/ml)	4.0 ± 2.0	3.7 ± 2.4	− 0.26 ± 0.49	0.521
C-peptide (ng/ml)	4.4 ± 6.0	4.1 ± 3.8	− 0.30 ± 0.14	0.681
HOMA-IR	1.6 ± 0.9	1.4 ± 0.8	− 0.21 ± 0.37	0.245
hsCRP (μg/ml)	5.3 ± 2.3	6.1 ± 3.9	0.81 ± 0.1.32	0.493

Data are presented as the mean ± SD.

Paired t-test was used to compare differences in participants before and after physical exercise.

*BMI* body mass index, *PBF* percent body fat, *SBP* systolic blood pressure, *DBP* diastolic blood pressure, *HR* heart rate, *VO*_*2*_*, max* maximum oxygen consumption, *TC* total cholesterol, *HDL* high-density lipoprotein, *LDL* low-density lipoprotein, *TG* triglyceride, *FBG* fasting blood glucose, *HbA1c* hemoglobin A1c, *HOMA-IR* Homeostatic Model Assessment for Insulin Resistance, *hsCRP* high-sensitivity C-reactive protein.

**Table 6:** Characteristics of all obese participants separated based on VO_2 max_ responders after exercise.

	Non-responders (N = 14)				Responders (N = 33)			
Markers	Pre	Post	Variation	*P* values	Pre	Post	Variation	*P* values
BMI (kg/m^2^)	33.18 ± 2.17	32.36 ± 4.53	− 0.82 ± 1.13	0.189	33.27 ± 2.76	32.80 ± 3.17	− 0.47 ± 0.84	0.373
Waist (cm)	107.57 ± 6.88	103.14 ± 9.63	− 4.43 ± 6.58	0.186	104.73 ± 10.95	102.80 ± 8.46	− 1.93 ± 2.49	0.259
Hip (cm)	111.21 ± 5.79	108.61 ± 6.99	− 2.60 ± 1.94	0.304	116.00 ± 7.64	113.25 ± 7.36	− 2.75 ± 4.14	0.239
PBF (%)	36.02 ± 3.52	34.48 ± 4.37	− 1.54 ± 1.32	0.160	37.15 ± 5.84	36.58 ± 5.75	− 0.57 ± 0.73	0.168
WBC10	6.44 ± 1.32	5.68 ± 1.17	− 0.76 ± 0.58	0.139	6.51 ± 1.94	6.68 ± 1.67	0.17 ± 0.22	0.679
SBP (mmHg)	115.71 ± 7.87	116.43 ± 4.76	0.72 ± 1.65	0.805	115.45 ± 9.34	114.73 ± 8.76	− 0.72 ± 1.92	0.437
DBP (mmHg)	77.14 ± 4.88	77.86 ± 2.67	0.71 ± 1.21	0.788	76.55 ± 5.22	75.00 ± 5.48	− 1.55 ± 2.13	0.846
Resting HR (beats/min)	83.86 ± 11.02	81.43 ± 14.11	− 2.43 ± 4.67	0.608	82.00 ± 6.71	75.55 ± 8.56	− 6.45 ± 4.39	0.042
VO_2_, max (ml/kg/min)	17.68 ± 2.97	17.18 ± 3.55	− 0.50 ± 0.28	0.462	17.89 ± 3.40	21.66 ± 4.70	3.77 ± 2.85	0.001
TC (mmol/l)	5.23 ± 1.14	5.34 ± 1.37	0.11 ± 0.29	0.767	4.79 ± 1.04	4.30 ± 0.93	− 0.49 ± 0.96	0.142
HDL (mmol/l)	1.24 ± 0.34	1.27 ± 0.40	0.03 ± 0.38	0.649	1.20 ± 0.37	1.21 ± 0.61	0.01 ± 0.32	0.906
LDL (mmol/l)	3.15 ± 1.30	3.62 ± 1.17	0.47 ± 0.42	0.384	2.96 ± 1.12	2.57 ± 0.66	− 0.39 ± 0.72	0.263
TG (mmol/l)	1.35 ± 0.89	1.37 ± 0.77	0.02 ± 0.19	0.963	1.16 ± 0.40	1.44 ± 0.90	0.28 ± 0.62	0.400
FBG (mmol/l)	6.37 ± 2.07	6.11 ± 2.13	− 0.26 ± 0.39	0.532	6.07 ± 1.07	5.85 ± 1.02	− 0.22 ± 0.46	0.158
HbA1c (%)	6.59 ± 1.49	6.34 ± 1.55	− 0.25 ± 0.27	0.134	6.34 ± 0.92	6.00 ± 0.71	− 0.34 ± 0.59	0.066
Insulin (ng/ml)	4.81 ± 2.12	3.61 ± 1.21	− 1.20 ± 1.09	0.156	5.40 ± 2.70	4.52 ± 2.11	− 0.88 ± 1.52	0.476
C-peptide (ng/ml)	11.32 ± 13.34	6.23 ± 7.31	− 5.09 ± 3.17	0.154	2.36 ± 1.15	2.98 ± 1.65	0.62 ± 0.42	0.290
HOMA-IR	1.72 ± 1.46	1.33 ± 1.15	− 0.39 ± 0.69	0.323	1.61 ± 0.80	1.14 ± 0.51	− 0.47 ± 0.94	0.205
hsCRP (μg/ml)	2.12 ± 1.67	1.62 ± 1.46	− 0.50 ± 0.21	0.178	3.85 ± 1.19	1.22 ± 0.59	− 2.63 ± 1.68	0.025

Data are presented as the mean ± SD.

*BMI* body mass index, *PBF* percent body fat, *SBP* systolic blood pressure, *DBP* diastolic blood pressure, *HR* heart rate, *VO*_*2*_*, max* maximum oxygen consumption, *TC* total cholesterol, *HDL* high-density lipoprotein, *LDL* low-density lipoprotein, *TG* triglyceride, *FBG* fasting blood glucose, *HbA1c* hemoglobin A1c, *HOMA-IR* Homeostatic Model Assessment for Insulin Resistance, *hsCRP* high-sensitivity C-reactive protein.

Our study into the effects of exercise on spexin levels found that, despite a general increase in spexin levels after exercise, some participants had decreased spexin levels after training. Participants were categorised as either responders or non-responders, where responders have increased spexin levels after exercise (Table 7) and showed that responders displayed greater amelioration in metabolic profile. Responders showed a significant increase in fitness as seen by amelioration of VO_2_ max with a concomitant decrease in HR, TC, HbA1c, HOMA-IR and hsCRP. Moreover, we observed a trend of decreased BMI, waist, insulin and LDL although this did not reach statistical significance (Table 7).

**Table 7:** Characteristics of all obese sorted based on spexin-responders after exercise.

	Non-responders (N = 19)				Responders (N = 28)			
Markers	Pre	Post	Variation	*P* values	Pre	Post	Variation	*P* values
BMI (kg/m^2^)	34.23 ± 2.83	33.58 ± 3.74	− 0.65 ± 1.13	0.131	33.55 ± 2.00	32.94 ± 1.91	− 0.63 ± 0.97	0.087
Waist (cm)	103.57 ± 6.81	101.86 ± 7.02	− 1.71 ± 2.49	0.079	109.80 ± 6.73	107.68 ± 4.79	− 2.12 ± 3.21	0.068
Hip (cm)	114.79 ± 8.35	113.30 ± 9.05	− 1.49 ± 4.08	0.197	113.57 ± 6.09	111.48 ± 5.73	− 2.09 ± 6.75	0.252
PBF (%)	40.15 ± 5.05	38.88 ± 6.30	− 1.27 ± 1.86	**0.037**	35.37 ± 3.94	34.58 ± 4.10	− 0.79 ± 1.90	0.129
WBC10	6.68 ± 2.01	6.61 ± 2.40	− 0.07 ± 0.49	0.830	6.75 ± 1.58	6.77 ± 1.61	0.02 ± 0.58	0.951
SBP (mmHg)	112.86 ± 9.51	112.86 ± 7.56	0.00 ± 3.25	1.000	120.00 ± 12.06	121.50 ± 7.67	1.50 ± 4.35	0.546
DBP (mmHg)	74.29 ± 5.35	76.43 ± 6.27	2.14 ± 4.09	0.510	76.67 ± 4.92	74.75 ± 5.38	− 1.92 ± 2.15	0.425
Resting HR (beats/min)	78.57 ± 9.81	78.86 ± 12.92	0.29 ± 2.02	0.857	85.58 ± 6.22	78.00 ± 10.62	− 7.58 ± 12.14	0.045
VO_2_, max (ml/kg/min)	16.74 ± 3.59	18.13 ± 3.97	1.39 ± 1.84	0.091	18.49 ± 2.80	21.05 ± 4.02	2.56 ± 3.88	0.035
TC (mmol/l)	4.96 ± 1.16	5.01 ± 1.22	0.05 ± 0.27	0.753	5.01 ± 1.07	4.47 ± 0.89	− 0.54 ± 1.03	0.018
HDL (mmol/l)	1.25 ± 0.41	1.34 ± 0.55	0.09 ± 0.23	0.111	1.08 ± 0.23	1.04 ± 0.23	− 0.04 ± 0.16	0.063
LDL (mmol/l)	3.18 ± 1.00	3.22 ± 1.09	0.04 ± 0.39	0.835	3.17 ± 1.15	2.83 ± 0.86	− 0.34 ± 0.88	0.191
TG (mmol/l)	1.21 ± 0.49	1.31 ± 0.80	0.10 ± 0.36	0.493	1.42 ± 0.59	1.63 ± 0.88	0.21 ± 0.67	0.318
FBG (mmol/l)	7.02 ± 2.82	6.62 ± 1.93	− 0.40 ± 1.07	0.418	7.26 ± 3.13	7.51 ± 3.44	0.25 ± 0.83	0.562
HbA1c (%)	7.12 ± 2.13	6.61 ± 1.32	− 0.51 ± 0.72	0.113	7.46 ± 2.15	6.72 ± 1.40	− 0.74 ± 1.06	0.003
Insulin (ng/ml)	4.34 ± 1.59	3.47 ± 1.57	− 0.87 ± 1.70	0.090	4.57 ± 2.59	3.62 ± 1.96	− 0.95 ± 2.39	0.088
C-peptide (ng/ml)	5.80 ± 7.82	4.92 ± 5.08	− 0.88 ± 2.17	0.465	6.26 ± 9.88	4.98 ± 5.70	− 1.28 ± 2.62	0.217
HOMA-IR	1.46 ± 0.78	1.03 ± 0.57	− 0.43 ± 0.52	**0.039**	1.45 ± 0.93	1.06 ± 0.69	− 0.39 ± 0.81	0.045
hsCRP (μg/ml)	4.11 ± 3.27	0.70 ± 0.34	− 3.41 ± 3.21	0.076	4.11 ± 2.69	1.15 ± 0.74	− 2.96 ± 2.57	< 0.001

Data are presented as the mean ± SD.

*BMI* body mass index, *PBF* percent body fat, *SBP* systolic blood pressure, *DBP* diastolic blood pressure, *HR* heart rate, *VO*_*2*_*, max* maximum oxygen consumption, *TC* total cholesterol, *HDL* high-density lipoprotein, *LDL* low-density lipoprotein, *TG* triglyceride, *FBG* fasting blood glucose, *HbA1c* hemoglobin A1c, *HOMA-IR* Homeostatic Model Assessment for Insulin Resistance, *hsCRP* high-sensitivity C-reactive protein.

## Discussion

The present study showed significantly decreased plasma spexin levels in obese individuals with or without diabetes compared with healthy normal-weight individuals. Plasma spexin levels were negatively correlated with adiposity markers and blood pressure in the whole population and the normal-weight group. Furthermore, multiple regression analysis revealed that blood pressure was the most predictive determinant of plasma spexin levels among these groups. However, in the obese without diabetes group, LDL and HOMA-IR were predictive variables for spexin levels, whereas in the obese with diabetes group, HDL levels could predict spexin circulating levels. Interestingly, spexin levels were significantly increased by regular physical exercise in both the obese groups, and plasma spexin levels might be considered as indicator of response to physical exercise.

Spexin, a 14-amino acid polypeptide, was first reported to suppress food intake in a goldfish model^7,19,20^. Chronic subcutaneous injection with spexin reduced food intake and led to weight loss in diet-induced obesity mice and rats^8^. Moreover, insulin induction by glucose stimulated spexin gene expression in goldfish hepatocytes and brain cells^21^. Taken together, these data highlight a key role of spexin in energy metabolism and weight regulation, with potential link to obesity and diabetes.

In agreement with these findings, the present study demonstrated that circulating levels of spexin were decreased with obesity and diabetes in adults and negatively correlated with adiposity markers (BMI and waist and hip circumference), DBP, systolic blood pressure (SBP) and lipid markers (LDL, TG and TC) but positively correlated with HDL levels. These findings are in line with those of previous reports that examined the role of spexin as a metabolic regulator of body weight and glucose homeostasis in obese adults and children or patients with type 2 diabetes mellitus and demonstrate that spexin could play an important role in obesity and diabetes^6^. A negative correlation was reported between spexin and FBG and HbA1c^6^. Unexpectedly, we found no clear association between circulating spexin levels and glycemic indicators in our adult population except a positive correlation with HOMA-IR in the obese without diabetes group and a negative correlation with HbA1c in the normal-weight group. This was further reflected by the observed comparable levels of circulating spexin in both the obese groups and within the obese with diabetes group with controlled and uncontrolled glycemic levels (Fig. 1). In contrast, in adolescent patients with obesity or diabetes, spexin levels did not vary significantly and there was no correlation with body composition or blood measurements, indicating that spexin may not act as a metabolic regulator in adolescents^9^.

These findings suggest that spexin might have different implications in obesity and diabetes depending on age as both of disorders are associated with age^22^. A recent study in healthy adult women reported that circulating spexin levels were negatively correlated with age, BMI, FBG and TG^23^. A decrease of spexin levels with age suggests a possible role of this peptide in ageing-related functions and disorders. However, despite the significantly older age of the obese with diabetes group in our study, we did not observe differences in spexin levels compared to the obese without diabetes group. This suggests that spexin pattern with age is blunted in diabetes status. Nevertheless, we cannot rule out the potential effects of treatment on spexin levels as study participants with diabetes were treated using antidiabetic as well as antihypertensive drugs, which might have contributed to the lack of difference of spexin between participants with and without diabetes. Very recently, Al-Daghri et al.^17^ reported increased circulating spexin levels in females with prediabetes but not in males after a 6-month self-monitored lifestyle modification programme. The same group has also reported an inverse association between spexin levels with fasting glucose in adult females with prediabetes, but not in males^10,17^. This might be due to a sexual dimorphism in spexin levels in relation to gender differences in glucose metabolism and insulin sensitivity due to sex steroids^24^. Categorisation of the obese without diabetes group into MHO and MUO groups revealed significantly lower spexin levels in the MUO group, which could not be explained by the increased glycaemic levels in the MUO. Therefore, it is crucial to elucidate the mechanisms of interaction between spexin and glucose/lipid metabolism.

Blood pressure increases with BMI^25^. The present study showed that blood pressure (SBP and DBP) was negatively correlated with spexin and DBP regardless of BMI and was an independent determinant of spexin levels in the whole population and the normal-weight group. This may indicate the involvement of spexin in the early development of cardiovascular disease (CVD) risk in apparently healthy individuals as reflected by decreased spexin levels when CVD risk factors (BMI, blood pressure, hyperlipidemia and hyperglycemia) are increased. In line with this hypothesis, a relationship between circulating spexin levels and markers of CVDs was previously reported in adolescents with obesity^26^, and spexin was proposed as an emerging marker for intervention and/or monitoring for CVD in children^27^. However, no significant correlation between spexin levels and blood pressure was found in adolescents^9^; therefore, further studies into the physiological roles and crosstalk of spexin with blood pressure are required, taking both age and BMI into consideration. It is worth noting that in our study, the negative association between spexin and lipids markers (TC, TG and LDL)—considered to be CVD risk factors—in the obese groups but not in the normal-weight groups endorses the protective role of spexin in metabolic disorders. In support of this hypothesis, spexin treatment reversed the dramatically increased hepatic levels of TG and total lipids in a diet-induced obesity mouse model without T2D^28^. The positive correlation and independent association of spexin with HDL further supports the protective role of spexin as HDL decreases with obesity. Furthermore, this relationship was previously suggested in individuals with metabolic syndrome^10^.

The present study shows for the first time that exercise increased plasma spexin levels and also improved cardiometabolic markers in obese individuals (Fig. 2). The pleiotropic beneficial effects of regular exercise are widely evidenced, especially in metabolic disorders. Indeed, regular exercise was reported in multiple studies, including ours, to improve glycemia, insulin sensitivity and cardiometabolic markers and reduce inflammation^29–31^. Interestingly, we showed that spexin levels were increased after exercise in both the obese groups with and without diabetes. We and others have previously reported greater beneficial effects of regular exercise in individuals without diabetes compared with those with diabetes based on other markers^30,32,33^.

The inhibitory effect of spexin on body weight in diet-induced obesity mice after chronic injection of spexin was suggested to be due to a reduced respiratory exchange ratio and increased locomotor activity^8^. Interestingly, in our study the increased levels of spexin observed after exercise were independent from BMI and diet, as only marginal changes in BMI were observed and no diet restrictions were prescribed with the exercise protocol. In line with this, spexin was reported among the BMI-independent changes observed over time in post-RYGB phase in morbidly obese youth, highlighting the fact that the clinical beneficial effects of RYGB may also involve spexin^14^.

On the other hand, spexin and its receptor, galanin receptor 2/3 (GALR2/3), are expressed in the central nervous system as well as various peripheral tissues, such as liver, heart, adipose tissue and skeletal muscle^34^. Since these tissues are involved in energy homeostasis and metabolic regulation, it was difficult to determine the source of increased circulating spexin after exercise in our study. Future studies are required to determine the tissue that facilitates spexin release in response to exercise to regulate body weight and food intake in obese patients with metabolic impairment.

Most previous studies using physical exercise intervention have reported the average effect of the exercise on the studied population. However, there is increased evidence that there is interindividual variability in response to exercise, as some individuals are responders who display improved metabolic markers, whereas others are non-responders and exhibit unchanged or worsened markers^35,36^. Several studies have reported a high prevalence of non-responders in diabetes with or without complications, supporting a relationship between diabetes and non-response^37,38^. Furthermore, MHO individuals showed higher levels of cardiorespiratory fitness than did MUO individuals, as reflected by our findings, suggesting that a healthier metabolic phenotype is linked, at least in part, to response to lifestyle factors^39^. This variability could be also associated with other biological factors, including genetics, which account for differences in body weight regulation in response to exercise^40,41^. Maximum oxygen consumption (VO_2_ max or VO_2_ peak) is the standard parameter of cardiorespiratory fitness and is widely used to assess the effectiveness of training and to estimate the prevalence of non-responders^36,42,43^.

Our findings showed significantly increased spexin levels in individuals who showed improvements in VO_2_ max regardless of other metabolic markers (Table 6 and Fig. 3). When we used spexin as a variable for classifying responders (with increased levels of spexin) versus non-responders, we found that more metabolic and inflammatory markers, including HR, VO_2_ max, TC, HbA1c, HOMA-IR and hsCRP, were improved in the responders (Table 7). This observed parallelism between VO_2_ max and spexin levels and improvement of metabolic markers in the responders highlights the potential value of spexin as a marker for exercise efficacy. Spexin, used independently or in a panel of markers, could help to tailor the intervention and thus maximise the beneficial effects of physical exercise in individuals with metabolic diseases, adapt fitness programmes for children and the elderly, or assess athletic performance. The inverse relationship between VO_2_ max and HR we have observed here is in agreement with the reported link between VO_2_ max and HRmax at submaximal exercise intensities^44^. Furthermore, the most important predictor of cardiovascular fitness and health as represented by VO_2_ max, was reported to be HR^45^.

To the best of our knowledge, the present study is the first to report the effect of exercise on spexin levels in obesity and diabetes. This study analysed a group of high-risk obese adults with diabetes who performed supervised moderate exercise as a lifestyle approach to improve global health without diet restriction. It is worth noticing that a poor adherence to recommended diet and intensive physical activity was reported in the country^46^. However, the present study had some limitations. The cross-sectional study design did not allow us to determine whether the decreased spexin levels contributed to the development of obesity and diabetes. Furthermore, the limited number of study participants did not allow generalisation of our findings. In addition, diabetes is associated with ageing, and it was challenging to find age-matched healthy normal-weight and obese controls. Moreover, our study was based on a single measurement of fasting spexin, and dietary intake was not controlled for in our study as well as the lack of detailed medication for our subjects, which may have affected spexin levels and the efficacy of physical exercise. The absence of normal-weight participants in our exercise protocol is another limitation which did not allow us to conclude on the status of spexin in those subjects. On the other hand, understanding spexin expression in adipose tissue and the effects of exercise in obese individuals with and without diabetes may elucidate the crosstalk between spexin levels and insulin resistance as well as its role in adipose tissue.

In conclusion, circulating spexin levels were decreased in human ‘diabesity,’ and physical exercise restored its levels concomitant with improved cardiometabolic markers. Plasma spexin levels may be an indicator of response to physical exercise. Further studies into the impact of physical exercise in mitigating the effects of metabolic stress linked to obesity and insulin resistance on spexin are required.

## Methods

### Study population, exercise protocol and anthropometric measurements

The current study recruited 185 adults. Written informed consents were obtained from all participants prior to taking part in the study that was conducted in line with the principles of the Declaration of Helsinki and was approved by our Institutional Review Board. The cohort was divided into three groups: normal-weight without diabetes (n = 50), obese without diabetes (n = 66) and obese with diabetes (n = 69). As we previously reported^47^, participants were excluded if they had regularly performed physical exercise within the last 6 months prior to enrolment to the study (being involved in any physical activity that is planned, structured, or repetitive at least once weekly) or had a history of major illness. Eligible participants for our 3-month supervised exercise programme were enrolled at the Fitness and Rehabilitation Centre of the Dasman Diabetes Institute^47,48^.

Briefly, Prior to exercise prescription each subject underwent the Cardiopulmonary exercise test “CPET” (COSMED, Quark, Italy) using electromagnetically braked cycle ergometer to measure the maximum heart rate (max HR) and maximum oxygen consumption (VO2 max). The termination criterion of 85% of age-predicted maximal heart rate (HR max) was used to stop the test. HR max was calculated as following: HR max = 220—age (year). Physical fitness was assessed thereafter to determine the muscle strength, endurance and flexibility by performing grip strength (dynamometer), push-ups upper body strength, sit ups and forward bending test (upper and lower body flexibility). As we previously reported^47^, the exercise program involved a combination of both resistance training with treadmill or cycling and moderate intensity aerobic exercise. Each exercise session included 10 min warming up and cooling down steps at 50–60% of max HR with 40 min of the mentioned program at 65–80% of max HR. Under the supervision of qualified fitness professionals at MFC, subjects exercised 3 times per week for a period of 3 months. They were monitored to maintain the recommended heart rates during the training. Anthropometric and whole-body composition measurements and exercise effectiveness was assessed at the end of the 3-month exercise program using similar fitness tests performed at baseline.

### Blood biochemical measurements

Venous peripheral blood samples were obtained at baseline and after the 3-month exercise programme and processed as previously reported^30^. Plasma biochemical and metabolic markers, including FBG and HbA1c, as well as lipid profile (TG, TC, LDL and HDL), were measured as previously reported^30^. hsCRP, insulin and C-peptide levels were measured by hsCRP ELISA (Biovendor, Asheville, NC, USA), insulin and ultrasensitive C-peptide ELISA, (Mercodia AB, Uppsala, Sweden). Plasma spexin levels were measured by ELISA (#EK-023-81, Phoenix Pharmaceuticals, Burlingame, California, USA). Plasma samples were diluted 1:10 prior to spexin measurement to fit within the detection range. All measurements were performed according to the manufacturers' instructions. Homeostatic Model Assessment of Insulin Resistance (HOMA-IR) index was calculated using the following formula: HOMA-IR = (glucose × insulin)/22.5.

### Statistical analysis

Statistical analyses were performed using SPSS software version 25.0 (SPSS Inc., Chicago, IL, USA) as we previously reported^47^. All descriptive statistics for variables in the study were reported as mean ± standard deviation. A Chi-square test was used for categorical variables, and a Wilcoxon nonparametric *t*-test was used for skewed variables. A two-way repeated measures ANOVA test with post hoc Bonferroni analysis was conducted to evaluate the effects of groups. A paired *t*-test was used to determine the significance of differences in means within each group before and after exercise. Correlations between variables were calculated using Spearman's correlation coefficient. Multivariate linear regression analysis was performed to examine the predictive effect of each factor. Values of *P* < 0.05 were considered statistically significant.

## Supplementary information

Supplementary information

## Data Availability

All generated data and resources are reported in this manuscript and there is no other data to be provided.

## References

[1] Roh E, Song DK, Kim MS (2016). Emerging role of the brain in the homeostatic regulation of energy and glucose metabolism. Exp. Mol. Med..

[2] van der Klaauw AA (2018). Neuropeptides in obesity and metabolic disease. Clin. Chem..

[3] Hirsch D, Zukowska Z (2012). NPY and stress 30 years later: the peripheral view. Cell Mol. Neurobiol..

[4] Meneguetti BT (2019). Neuropeptide receptors as potential pharmacological targets for obesity. Pharmacol. Ther..

[5] Mirabeau O (2007). Identification of novel peptide hormones in the human proteome by hidden Markov model screening. Genome Res..

[6] Lv SY, Zhou YC, Zhang XM, Chen WD, Wang YD (2019). Emerging roles of NPQ/spexin in physiology and pathology. Front. Pharmacol..

[7] Wong MK (2013). Goldfish spexin: solution structure and novel function as a satiety factor in feeding control. Am. J. Physiol. Endocrinol. Metab..

[8] Walewski JL (2014). Spexin is a novel human peptide that reduces adipocyte uptake of long chain fatty acids and causes weight loss in rodents with diet-induced obesity. Obesity.

[9] Hodges SK, Teague AM, Dasari PS, Short KR (2018). Effect of obesity and type 2 diabetes, and glucose ingestion on circulating spexin concentration in adolescents. Pediatr. Diabetes.

[10] Al-Daghri NM (2018). Spexin levels are associated with metabolic syndrome components. Dis. Markers.

[11] Toll L (2012). Peptides derived from the prohormone proNPQ/spexin are potent central modulators of cardiovascular and renal function and nociception. FASEB J..

[12] Grewen KM, Light KC, Mechlin B, Girdler SS (2008). Ethnicity is associated with alterations in oxytocin relationships to pain sensitivity in women. Ethn. Health.

[13] Heinonen S (2017). Mitochondria-related transcriptional signature is downregulated in adipocytes in obesity: a study of young healthy MZ twins. Diabetologia.

[14] Kumar S, Hossain MJ, Inge T, Balagopal PB (2018). Roux-en-Y gastric bypass surgery in youth with severe obesity: 1-year longitudinal changes in spexin. Surg. Obes. Relat. Dis..

[15] Zhang N, Bi S (2018). Effects of physical exercise on food intake and body weight: role of dorsomedial hypothalamic signaling. Physiol. Behav..

[16] Shin MS (2003). Treadmill exercise suppresses diabetes-induced increment of neuropeptide Y expression in the hypothalamus of rats. Neurosci. Lett..

[17] Al-Daghri NM (2019). Favorable changes in fasting glucose in a 6-month self-monitored lifestyle modification programme inversely affects spexin levels in females with prediabetes. Sci. Rep..

[18] National Cholesterol Education Program Expert Panel on Detection, & Treatment of High Blood Cholesterol in Adults (2002). Third report of the National Cholesterol Education Program (NCEP) expert panel on detection, evaluation, and treatment of high blood cholesterol in adults (adult treatment panel III) final report. Circulation.

[19] Wu H (2016). Ya-fish (Schizothorax prenanti) spexin: identification, tissue distribution and mRNA expression responses to periprandial and fasting. Fish Physiol. Biochem..

[20] Li S (2016). Molecular cloning and functional characterization of spexin in orange-spotted grouper (*Epinephelus coioides*). Comp. Biochem. Physiol. B Biochem. Mol. Biol..

[21] Ma A (2017). Dual role of insulin in spexin regulation: functional link between food intake and spexin expression in a fish model. Endocrinology.

[22] Tzanetakou IP, Katsilambros NL, Benetos A, Mikhailidis DP, Perrea DN (2012). "Is obesity linked to aging?": adipose tissue and the role of telomeres. Ageing Res. Rev..

[23] Lin CY (2018). Circulating spexin levels negatively correlate with age, BMI, fasting glucose, and triglycerides in healthy adult women. J. Endocr. Soc..

[24] Macotela Y, Boucher J, Tran TT, Kahn CR (2009). Sex and depot differences in adipocyte insulin sensitivity and glucose metabolism. Diabetes.

[25] Vaneckova I (2014). Obesity-related hypertension: possible pathophysiological mechanisms. J. Endocrinol..

[26] Kumar S, Hossain MJ, Javed A, Kullo IJ, Balagopal PB (2018). Relationship of circulating spexin with markers of cardiovascular disease: a pilot study in adolescents with obesity. Pediatr. Obes..

[27] Daniels SR (2019). Promoting cardiovascular health in early childhood and transitions in childhood through adolescence: a workshop report. J. Pediatr..

[28] Ge JF, Walewski JL, Anglade D, Berk PD (2016). Regulation of hepatocellular fatty acid uptake in mouse models of fatty liver disease with and without functional leptin signaling: roles of NfKB and SREBP-1C and the effects of spexin. Semin. Liver Dis..

[29] Lancaster GI, Febbraio MA (2014). The immunomodulating role of exercise in metabolic disease. Trends Immunol..

[30] Khadir A (2018). Physical exercise enhanced heat shock protein 60 expression and attenuated inflammation in the adipose tissue of human diabetic obese. Front. Endocrinol. (Lausanne).

[31] Ivy JL (1997). Role of exercise training in the prevention and treatment of insulin resistance and non-insulin-dependent diabetes mellitus. Sports Med..

[32] Khadir A (2018). Fetuin-A levels are increased in the adipose tissue of diabetic obese humans but not in circulation. Lipids Health Dis..

[33] Blaak EE, van Aggel-Leijssen DP, Wagenmakers AJ, Saris WH, van Baak MA (2000). Impaired oxidation of plasma-derived fatty acids in type 2 diabetic subjects during moderate-intensity exercise. Diabetes.

[34] Kim DK (2014). Coevolution of the spexin/galanin/kisspeptin family: spexin activates galanin receptor type II and III. Endocrinology.

[35] Bonafiglia JT (2016). Inter-individual variability in the adaptive responses to endurance and sprint interval training: a randomized crossover study. PLoS ONE.

[36] Bouchard C, Rankinen T (2001). Individual differences in response to regular physical activity. Med. Sci. Sports Exerc..

[37] Chmelo EA (2015). Heterogeneity of physical function responses to exercise training in older adults. J. Am. Geriatr. Soc..

[38] Gardner AW, Parker DE, Montgomery PS, Blevins SM (2014). Diabetic women are poor responders to exercise rehabilitation in the treatment of claudication. J. Vasc. Surg..

[39] Ortega FB (2018). Role of physical activity and fitness in the characterization and prognosis of the metabolically healthy obesity phenotype: a systematic review and meta-analysis. Prog. Cardiovasc. Dis..

[40] Bouchard C (1994). The response to exercise with constant energy intake in identical twins. Obes. Res..

[41] Stubbe JH (2006). Genetic influences on exercise participation in 37,051 twin pairs from seven countries. PLoS ONE.

[42] Whipple MO (2018). Variability in individual response to aerobic exercise interventions among older adults. J. Aging Phys. Act..

[43] Bohm A, Weigert C, Staiger H, Haring HU (2016). Exercise and diabetes: relevance and causes for response variability. Endocrine.

[44] Garber CE (2011). American College of Sports Medicine position stand. Quantity and quality of exercise for developing and maintaining cardiorespiratory, musculoskeletal, and neuromotor fitness in apparently healthy adults: guidance for prescribing exercise. Med. Sci. Sports Exerc..

[45] Grant CC, Murray C, Janse van Rensburg DC, Fletcher L (2013). A comparison between heart rate and heart rate variability as indicators of cardiac health and fitness. Front. Physiol..

[46] Serour M, Alqhenaei H, Al-Saqabi S, Mustafa AR, Ben-Nakhi A (2007). Cultural factors and patients' adherence to lifestyle measures. Br. J. Gen. Pract..

[47] Kavalakatt S (2019). Urocortin 3 levels are impaired in overweight humans with and without type 2 diabetes and modulated by exercise. Front. Endocrinol..

[48] Tiss A (2014). Immunohistochemical profiling of the heat shock response in obese non-diabetic subjects revealed impaired expression of heat shock proteins in the adipose tissue. Lipids Health Dis..

