# Supplementary material for: Spexin as an indicator of beneficial effects of exercise in human obesity and diabetes
Source: Sci Rep. 2020 Jun 30;10:10635. doi: 10.1038/s41598-020-67624-z (PMC7327065; doi:10.1038/s41598-020-67624-z)
Supplement: Supplementary file 1 — Supplementary information [file 41598_2020_67624_MOESM1_ESM.docx]

**Spexin as an indicator of beneficial effects of exercise in human obesity and diabetes**

**Abdelkrim Khadir^1^, Sina Kavalakatt^1^, Dhanya Madhu^1^, Sriraman Devarajan^2^, Jehad Abubaker^1^, Fahd Al-Mulla^2^ and Ali Tiss^1^***

^1^ Biochemistry and Molecular Biology department, Research Division, Dasman Diabetes Institute, Kuwait

^2^ Research Division, Dasman Diabetes Institute, Kuwait

**Supplementary Tables**

***Correspondence**

Ali Tiss, PhD

Biochemistry and Molecular Biology department,

Research Division, Dasman Diabetes Institute,

P.O. Box1180, Dasman,15462, Kuwait

Tel: +96522242999 ext. 3553

E-mail: [ali.tiss@dasmaninstitute.org](mailto:ali.tiss@dasmaninstitute.org)

ORCID ID: 0000-0002-3024-5370

**Table S1: Characteristics of the obese without diabetes group based on MHO and MUO classification**

|  | MHO | MUO | P Values |
| --- | --- | --- | --- |
| Gender (M/F) | 7/10 | 22/27 | 0.792 |
| Age (years) | 42.53±12.24 | 43.53±13.33 | 0.779 |
| BMI (kg/m^2^) | 33.03±2.29 | 35.36±3.01 | **0.016** |
| Waist (cm) | 103.25±12.61 | 106.93±10.18 | 0.370 |
| Hip (cm) | 113.29±7.53 | 118.78±8.49 | 0.045 |
| PBF (%) | 38.30±4.28 | 39.17±5.77 | 0.588 |
| WBC10 | 5.89±1.60 | 6.52±1.76 | 0.182 |
| SBP (mmHg) | 114.50±7.54 | 121.98±10.30 | **0.025** |
| DBP (mmHg) | 73.50±6.22 | 78.93±6.85 | **0.048** |
| Resting HR (beats/min) | 82.25±8.68 | 77.78±11.53 | **0.037** |
| VO_2_, max (ml/kg/min) | 19.14±4.48 | 16.87±3.59 | 0.094 |
| TC (mmol/l) | 5.19±0.99 | 5.05±0.95 | 0.608 |
| HDL (mmol/l) | 1.46±0.29 | 1.18±0.34 | **0.003** |
| LDL (mmol/l) | 3.35±0.82 | 3.29±0.91 | 0.779 |
| TG (mmol/l) | 0.97±0.44 | 1.41±0.69 | **0.004** |
| FBG (mmol/l) | 4.98±0.29 | 5.66±1.10 | **0.001** |
| HbA1c (%) | 5.31±0.56 | 5.89±1.15 | **0.008** |
| Insulin (ng/ml) | 3.70±1.50 | 3.93±2.79 | 0.698 |
| C-peptide (ng/ml) | 5.08±3.60 | 5.65±7.63 | 0.709 |
| HOMA-IR | 0.83±0.33 | 1.01±0.73 | 0.228 |
| hsCRP (μg/ml) | 5.18±3.43 | 6.20±3.67 | 0.499 |

**Table S2: Characteristics of the obese with diabetes group based on HbA1c levels**

|  | HbA1c < 6.5 | HbA1c ≥ 6.5 | P value |
| --- | --- | --- | --- |
| Gender (M/F) | 11/7 | 25/26 | 0.376 |
| Age (years) | 53.65±10.25 | 51.61±9.17 | 0.400 |
| BMI (kg/m^2^) | 34.82±2.88 | 34.04±2.44 | 0.252 |
| Waist (cm) | 110.33±8.21 | 110.15±8.82 | 0.934 |
| Hip (cm) | 117.08±12.33 | 113.83±12.02 | 0.091 |
| PBF (%) | 38.22±15.06 | 37.74±4.77 | 0.478 |
| WBC10 | 7.36±1.54 | 7.45±1.91 | 0.811 |
| SBP (mmHg) | 118.95±12.86 | 123.75±10.80 | 0.153 |
| DBP (mmHg) | 77.37±7.33 | 79.17±5.91 | 0.339 |
| Resting HR (beats/min) | 79.84±15.26 | 83.22±12.59 | 0.390 |
| VO_2_, max (ml/kg/min) | 14.95±4.03 | 15.42±4.11 | 0.659 |
| TC (mmol/l) | 4.97±1.12 | 4.95±1.16 | 0.944 |
| HDL (mmol/l) | 1.17±0.39 | 1.18±0.39 | 0.922 |
| LDL (mmol/l) | 3.15±0.96 | 2.94±1.34 | 0.427 |
| TG (mmol/l) | 1.44±0.57 | 1.94±1.28 | **0.011** |
| FBG (mmol/l) | 5.29±2.39 | 9.98±3.53 | **<0.001** |
| HbA1c (%) | 6.01±0.32 | 9.11±1.52 | **<0.001** |
| Insulin (ng/ml) | 4.04±2.17 | 4.00±2.03 | 0.950 |
| C-peptide (ng/ml) | 2.73±2.10 | 5.02±6.59 | **0.045** |
| HOMA-IR | 1.18±0.65 | 1.61±0.94 | **0.049** |
| hsCRP (μg/ml) | 3.08±1.67 | 6.17±4.59 | **0.006** |

**Table S3: Spearman correlation of VO_2_ max, HR and circulating Spexin with characteristics of individuals enrolled in exercise protocol (n=47).**

|  | Pre | | | Post | | |
| --- | --- | --- | --- | --- | --- | --- |
|  | **HR** | **VO_2_ max** | **Spexin** | **HR** | **VO_2_ max** | **Spexin** |
| BMI (kg/m^2^) | 0.179 | -0.127 | 0.269 | 0.320 | **-0.376*** | -0.139 |
| Waist (cm) | 0.230 | 0.115 | 0.099 | 0.080 | 0.161 | -0.056 |
| Hip (cm) | 0.185 | -0.233 | 0.124 | -0.165 | 0.001 | **-.453^*^** |
| PBF (%) | 0.010 | **-.403^**^** | 0.271 | 0.353 | -0.302 | -0.048 |
| WBC10 | 0.263 | -0.271 | 0.208 | 0.174 | 0.200 | 0.179 |
| SBP (mmHg) | 0.294 | -0.061 | -0.104 | 0.176 | 0.118 | -0.168 |
| DBP (mmHg) | **.329^*^** | 0.078 | -0.089 | -0.024 | 0.140 | -0.119 |
| Resting HR (beats/min) | 1.000 | -0.009 | 0.065 | 1.000 | **-0.417*** | -0.050 |
| VO_2_, max (ml/kg/min) | -0.009 | 1.000 | -0.006 | **-0.417*** | 1.000 | 0.292 |
| TC (mmol/l) | -0.012 | 0.237 | -0.233 | -0.177 | -0.087 | -0.080 |
| HDL (mmol/l) | -0.003 | -0.018 | 0.178 | 0.164 | -0.226 | 0.147 |
| LDL (mmol/l) | -0.033 | 0.274 | -0.210 | -0.214 | -0.120 | -0.142 |
| TG (mmol/l) | -0.129 | -0.145 | **-.348^*^** | 0.225 | 0.022 | -0.117 |
| FBG (mmol/l) | 0.002 | **-.342^*^** | -0.078 | -0.125 | -0.040 | -0.145 |
| HbA1c (%) | 0.087 | -0.200 | -0.139 | -0.086 | 0.241 | -0.164 |
| Insulin (ng/ml) | 0.101 | 0.227 | 0.002 | -0.283 | 0.120 | **-.358^*^** |
| C-peptide (ng/ml) | **.417^*^** | -0.001 | -0.109 | 0.177 | 0.120 | -0.127 |
| HOMA-IR | 0.158 | 0.008 | -0.052 | -0.211 | 0.145 | **-.487^**^** |
| hsCRP (μg/ml) | 0.197 | -0.164 | 0.309 | -0.211 | 0.145 | **-.490^**^** |
| Spexin (ng/ml) | 0.065 | -0.006 | 1.000 | -0.050 | 0.292 | 1.000 |

*BMI (Body mass index), PBF (percent body fat), SBP (systolic blood pressure), DBP (diastolic blood pressure), HR (heart rate), VO_2_, max (maximum oxygen consumption), TC (total cholesterol), HDL (high-density lipoprotein), LDL (low-density lipoprotein), TG (triglyceride), FBG (fasting blood glucose), HbA1c (hemoglobin A1c), HOMA-IR (Homeostatic Model Assessment for Insulin Resistance), and hsCRP (high-sensitivity C-reactive protein). Significance of spearman correlation are as follows *: p<0.05, **:p<0.01.*
